# A Memoir of the Life and Character of Philip Syng Physick, M. D.

**Published:** 1840-11

**Authors:** 


### REVIEWS.

---

Art. V.—1. *A Memoir of the Life and Character of Philip Syng Physick, M. D.* By J. Randloph, M. D., Lecturer on Surgery, Member of the American Philosophical Society, etc., etc. Philadelphia: T. K. & P. G. Collins. 1839. 8vo. pp. 114.

2. *Necrological Notice of Dr. Philip Syng Physick; delivered before the American Philosophical Society, May 4th,* 1838. By W. E. Horner, M. D., Professor of Anatomy in the University of Pennsylvania, etc., etc. Philadelphia: Haswell, Barrington & Haswell. 1838. 8vo. pp. 32.

The biography of Dr. Physick possesses no uncommon interest, if we estimate it by the incidents which it affords. Yet it is a biography which, on account of the celebrity of the individual, will be extensively read, and which is calculated to exercise a most excellent influence upon the young men of the profession of which he was for many years the acknowledged head and centre in America. Dr. Physick was reputed, and we believe justly, a great man—as a Surgeon pre-eminently great; but his greatness was of a kind which does not oppress or alarm the beholder, but rather inspires him with a hope that he may attain to something like it, because it was the result of industry long directed to a single great object. It was a greatness, too, which we may admire without any painful abatement on the score of mingling weaknesses. It was alloyed by no base quality. The lustre of his long life, passed before the public eye, was tarnished by no vice. Still, in contemplating his character, we find nothing extraordinary about him, but his extraordinary skill in surgery, and this he owed to a life of the greatest pains-taking. His character was cast in the common mould, and his superiority was the growth of time, and appplication, and the sound moral culture which he received from exemplary parents, especially a pious mother. It is one, therefore, which we may study with decided profit, for it is of such worthies that it is especially true, that

"The lives of great men all remind us
We may make our lives sublime,
And departing leave behind us
Footsteps on the sands of time;
Footsteps, that perhaps another,
Sailing o'er life's solemn main,
A forlorn and shipwrecked brother,
Seeing, shall take heart again."

Dr. Physick was born in Philadelphia, on the 7th of July, 1768, of respectable parents. His father, an Englishman by birth, was a man of strong, sagacious mind, and strict integrity of principle. His mother was a pious and highly estimable woman, with a sound judgment, and great decision of character, in which qualities she was strongly resembled by her son. Having taken the degree of Bachelor of Arts in the collegiate department of the University of Pennsylvania, it became necessary that he should make choice of a profession. The mechanical turn for which he became so remarkable in his profession had early shown itself in his character, and he declared his preference for the trade of a silversmith, in which, fortunately for the interests of science and humanity, he was over-ruled by an inflexible father, who placed him under the care of Dr. Adam Kuhn to study medicine. It is said that Mr. Physick was led to this choice by the skill which his son displayed in applying the bandage and dressings to his finger, which he had wounded whilst handling a knife. But his son seems never to have lost, even amid his most brilliant successes, this early inclination to mechanical pursuits. Dr. Horner relates, that

"Even within a short time of his death, in reflecting upon the events of his life, such as his anxieties for the health of others, his professional excitements, and the decrepitude and ravages made on his own constitution by disease, he regretted that he had not been indulged in his love for the business of a silversmith: his impression was that in securing to himself health and tranquil employment, his life would have been much happier, and a very sufficient measure of success would have attended his efforts."

Cullen's first Lines of the Practice of Physic was the book of the highest reputation at that day, and was handed to him by his venerated preceptor with such commendations that he was induced to commit it to memory—a feat, we presume, which has not often been performed since, and which it may safely be predicted will never occur again. The first experience of the young surgeon gave no promise of that coolness and self-control to which he afterwards attained, for the boiling of a skeleton, which he was called to witness, is reported to have excited in him so strong a disgust to the profession of medicine, that he renewed his entreaties to his father to permit him to become a silversmith. On another occasion, while witnessing an amputation, at the Pennsylvania Hospital, he became so sick that his father, who was present, was obliged to lead him from the amphitheatre before the operation was concluded. These incidents in the life of the Father of American Surgery will, doubtless, console and encourage many a pupil who may be compelled, by a like infirmity, to retreat from the first operation of his first winter, amid the ridicule of his less sensitive class-mates.

Dr. Physick having continued to prosecute his studies under the direction of Dr. Kuhn for three years, during which time he also attended the medical lectures delivered in the University of Pennsylvania, embarked for Europe in November, 1788, when twenty years of age, for the purpose of completing his education in the great schools and hospitals of London and Edinburgh. In visiting Europe, his sole purpose was the acquisition of medical knowledge. No other object shared with this in his attention. No pleasure was seductive enough to lure him from his studies. He was fortunate in securing the services of John Hunter as a preceptor, and more fortunate in imbibing the spirit of his great master. Mr. Hunter, it is well known, was no great student of the writings of others, but he was indefatigable in the industry with which he pursued his original reseaches with the dissecting knife; and it was under his auspices that Dr. Physick acquired that minute knowledge of anatomy to which he afterwards owed his unrivalled reputation as a surgeon. The following anecdote related by Dr. Horner, is strikingly characteristic of Mr. Hunter, and shows the bias which determined the future career of his pupil.

"The affair being settled that young Mr. Physick was to study under him, the elder Mr. Physick said, 'Well, sir, I presume some books will be required for my son, I will thank you to mention them that I may get them.' 'Here, sir,' says Mr. Hunter, 'follow me; I will show the books your son has to study.' Mr. Hunter led the way from his study to his dissecting-room, and entering it pointed to several dead bodies; 'These are the books,' said he, 'which your son will learn under my direction; the others are fit for very little.' The impression made on the mind of Dr. Physick was durable; he never forgot the remark, especially after committing to memory Cullen's First Lines, as he had done but a short time before in Philadelphia."

From this period forward, Dr. Physick seems to have read little of books, but, like his preceptor, to have relied upon his own personal observation in the dissecting-room, and at the bed side of the sick, for a knowledge of the nature and treatment of disease. Two years after entering upon his apprenticeship with Mr. Hunter, we find him elected house surgeon to St. George's Hospital, which post he obtained over a number of competitors, chiefly through the influence of Mr. Hunter. His success very naturally caused some dissatisfaction among the disappointed applicants, who conceived that their claims for the situation were stronger than those of the young foreigner.

"In consequence of this, Dr. Physick clearly perceived that they evinced an uncommon share of curiosity respecting the manner in which he discharged his duties, and that they were disposed to scrutinize his actions with the greatest strictness. A short period after commencing his services, a patient was admitted into the hospital who had had the misfortune to dislocate his shoulder; the head of the humerus was thrown downward, and loged in the axilla. Fortunately, the accident was quite recent. It so happened that at the time the man was admitted the whole class were in attendance at the house. They, of course, were exceedingly anxious to witness the manner in which the reduction would be effected, and Dr. Physick was perfectly well aware that his method of restoring the bone to its natural situation would be subjected to severe criticism. He directed the patient to be seated upon a high chair; he then proceeded to examine the injured shoulder very particularly, and questioned the man as to the manner in which the accident had occurred. Whilst making these inquiries he placed his left hand in the axilla, and taking hold of the lower end of the humerus with his right hand, he made all the extension in his power; he then suddenly depressed the elbow of the patient to the side of his body, and in so doing, dislodged the head of the bone, which glided instantaneously into the glenoid cavity, very much to his own delight, and doubtless also to the perfect satisfaction of the class."—*Memoir, p.* 24.

In 1791 he received his diploma from the Royal College of Surgeons in London, and after spending a winter in the University of Edinburgh took the degree of Doctor of Medicine in 1792. So high an opinion had Mr. Hunter by this time conceived of his qualifications, that he invited him to take up his residence with him, to become an inmate of his house, and to establish himself permanently in London to pursue the practice of his profession. An apprehension that the climate and atmosphere of that city might injure his health, is said to have been the principal cause in determining him to decline so an advantageous an offer. Returning home the same year, he commenced the practice of physic in Philadelphia, with only two shillings and sixpence in his pocket. His father, having qualified him for usefulness and distinction, said to him, "My son, you have cost me much money; you have now an outfit, learn to take care of yourself." But with all his advantages, as well of person as of education, he was slow in getting into business, and was so discouraged after more than a year of fruitless probation, that he declared he would have sold the fee simple of his profession for a thousand pounds, have retired into the country, and never again have felt a pulse in the capacity of a physician. "I walked the pavements of Philadelphia," said he to Dr. Caldwell, "after my return from Europe, for nearly three years, without making as much by my practice as put soles on my shoes."* In this state of affairs he felt constrained to resort to the following expedient, which was the beginning of the great business to which he at length attained.

* Caldwell's Eulogy of Physick.

"Many of his hours," says Dr. Horner, "were spent, by invitation in a fine library belonging to a Mr. Priestman. He on one occasion said to Mr. P., 'I should feel much more happy, if I had something like a certain livelihood: if your family and some others would give me, at any rate, twenty dollars a year, each, for acting as their physician, I should be satisfied.' Mr. Priestman had a very high opinion of him and replied immediately, 'Well, Doctor, I agree to that:' and being a man of influence, he interested several other families in the same way."

After all, it is probable the Doctor indulged in a little pardonable exaggeration to his friend, Dr. Caldwell, in describing the difficulties attending his early practical career, since we find him in 1793, one year after his return from Europe, acting as physician to the Bush Hill Hospital, erected for the accommodation of indigent persons laboring under yellow fever, and one year later risen to be a prescribing physician in the Philadelphia Dispensary, and one of the surgeons to the Pennsylvania Hospital. It is true that these were offices of no immediate profit, but public confidence, according to one of his biographers, kept pace with this promotion, as was exhibited by his practice increasing with no ordinary rapidity. And after his second term of service at this Hospital, in 1798, his zeal and fidelity were recognized in the presentation of some elegant pieces of silver plate by the Board of Managers. Of the firmness and moral courage displayed by him in the midst of this epidemic; of the self-sacrificing spirit which animated him, in common with his medical brethren, whilst others were flying from the danger, the following anecdote related by Dr. Horner affords a striking example:

"Dr. Physick having offered his services, was chosen physician of the Bush Hill Hospital. He left his lodgings in town, entered immediately upon his new duties, and continued in the exercise of them till the disease had passed away. While on this duty, a disposition to insubordination and riot having been exhibited by the inmates of the hospital, he was appointed alderman by Governor Mifflin, so as to meet any emergency with the promptest attention and vigor. An explanation of this civil magistracy may be given, by stating that such was the panic created by the yellow fever, that no ordinary civil officer could be found hardy enough, to enter the hospital to enforce order. I have repeatedly heard the Doctor say, that when he was sworn in by the Mayor (Matthew Clarkson,) the latter held off from the precincts of the hospital, and from Dr. Physick, at the greatest distance compatible with hearing, and was happy to be off as soon as possible, when the ceremony was over."

His constant exposure to the poison brought on an attack of the fever; and in 1797 he experienced a second attack, during which, it is stated by Dr. Rush, Dr. Dewees took from him one hundred and seventy-six ounces of blood. The disease brought him near to the grave.

"He told me," says Dr. Horner, "that, as he lay ill, he could hear through his windows, which were open, a stout blacksmith, who lived near, inquire daily of his black waiter, is your master dead yet?" To which the monotonous response of "No!" was as often given. When the Doctor recovered sufficiently to leave the house, he inquired after the blacksmith who had so frequently saluted his ears with this disagreeable question. He found that the blacksmith himself, had in the mean time been one of the victims of the epidemic."

He made many post mortem examinations during the prevalence of the epidemic, and arrived at the conclusion of which so much has since been made by Broussais, that yellow fever has its origin in gastritis. The establishment of the fact, that the disease was of an inflammatory character was a most important service for the profession, as it confirmed the superiority of the anti-phlogistic treatment over the opposite which had generally prevailed. It may be pronounced a great discovery, and it certainly confers no little renown upon

Dr. Physick that he was instrumental in bringing about one of the most salutary revolutions known in the history of practical medicine. Nor did he render a much less important service in contending with Dr. Caldwell, then, like himself a young practitioner in the city of Philadelphia, against the high authority of Dr. Rush, for the non-contagious character of this fever.

In 1797 was performed his first operation for stone in the bladder, which derives interest from the circumstance, that in making it his gorget divided the internal pudic artery. The hemorrhage from the wounded vessel was alarmingly profuse. Dr. Randolph describes his procedure in the emergency.

"He immediately compressed the trunk of the artery with the fore finger of his left hand, and then passed the point of a tenaculum under it, a ligature was then cast round it and firmly tied. This of course arrested the hemorrhage, but the ligature included along with the artery a considerable portion of the adjacent flesh. In order to obviate this inconvenience Dr. Physick subsequently contrived his celebrated forceps and needle for the purpose of carrying a ligature under the pudic artery."

He was married in 1800 to Miss Emlen, the daughter of a gentleman of learning, distinction and wealth, who belonged to the society of Friends. Mrs. Physick died in 1820, leaving four children, now alive—two sons and two daughters. In the same year, he was invited by a number of the medical students of the University of Pennsylvania to lecture to them on Surgery. He acceded to their wishes, and in doing so, how great he felt the resposibility to be, and how zealously he labored to acquit himself well, the following anecdote related by Dr. Randolph, will testify:

"After preparing the lecture introductory to his course, he committed it to memory. Among the persons invited to be present at its delivery was his valued friend, Dr. Rush. The scene was a trying one to Dr. Physick. It was the first time he had ever publicly addressed an audience. At the close of the lecture, Dr. Rush stepped up to him and gave him his hand, and congratulated him upon his success. He then said to him emphatically, 'Doctor, that will do—that will do. You need not be apprehensive as to the result of your lecturing—I am sure you will succeed.' Dr. Physick never forgot Dr. Rush's kind manner to him on this occasion. He assured me that it exerted a considerable influence in strengthening and confirming his resolutions to persevere.

It was not until five years afterwards that he was called to the chair of Surgery in the University, to aid his friend Rush in carrying that school to a pitch of popularity which few institutions have exceeded. Dr. Randolph says:

"At the period when Dr. Physick commenced his professional career the organization of the medical department in the University of Pennsylvania was so imperfect, that the chairs of Anatomy and Surgery were combined, and the duties of teaching both branches devolved upon one Professor. In order to remedy this acknowledged deficiency, in the year 1805, the chair of Surgery was made distinct from that of Anatomy, and Dr. Physick was elected, I believe unanimously, Professor of Surgery."

In the mean time he had been elected Surgeon Extraordinary in the Philadelphia Alms House, and a member of the American Philosophical Society.

At this period of his life his habits were remarkably laborious.

"He has frequently told me," says Dr. Randolph, "that it was his custom, throughout the winter months, to rise at four o'clock in the morning. This hour being too early to disturb a servant, he was obliged to arrange his own fire. He would then sit down to his desk, and prepare his lecture for the day; after which he would dress himself, and then take his breakfast, and leave his house between eight and nine o'clock, in order to attend to a most extensive and laborious practice. In addition to this, he discharged his duties as Surgeon to the Pennsylvania Hospital, and to the Alms House Infirmary. He used often to remark, that in order to obtain entire success as a practitioner of medicine, it was necessary to work hard. It will be conceded that no portion of his success ever came to him gratuitously; on the contrary, he made laborious exertions to obtain it."

In July, 1819, he resigned the chair of Surgery, and was appointed to that of Anatomy, made vacant in the preceding November by the death of Dr. Dorsey, his favorite nephew. We always regarded this transfer as an unfortunate one both for Dr. Physick and the institution, and we find that such were his own impressions, and those of his most intimate friends. Dr. Randolph says:

"It was always a source of deep regret with Dr. Physick's immediate family and friends, that his comforts in the evening of his days, and whilst laboring under physical infirmities, should be so greatly interrupted by translating him from the chair of Surgery to that of Anatomy. We had positive assurances from himself that the change was contrary to his own wishes and inclination."

In 1821, he was appointed Consulting Surgeon to the Institution for the Blind; in 1822, the Phrenological Society of Philadelphia elected him its President; he was chosen President of the Philadelphia Medical Society in 1824, which office he held to the time of his death; in 1825 he was appointed a Member of the Royal Academy of Medicine of France; and in 1831, he was unanimously elected *Emeritus* Professor of Surgery and Anatomy, having in consequence of declining health retired from the active duties of the University.

In October 1831, he performed the operation of lithotomy on Chief Justice Marshall. The following account of it by Dr. Randolph is interesting:

"The case was attended with singular interest, in consequence of the exalted position of the patient, his advanced age, and the circumstance of there being upward of one thousand calculi taken from his bladder. It is well known that for several years previous to this period, Dr. Physick had declined performing extensive surgical operations. He felt somewhat reluctant to operate on Chief Justice Marshall, and offered to place the case in my hands. Taking all the circumstances into consideration, and knowing well that this would be the last time that he would ever perform a similar operation, I felt desirous that he should finish with so distinguished an individual, and accordingly urged him to do it himself. Upon the day appointed, the Doctor performed the operation with his usual skill and dexterity. I do not think I ever saw him display greater neatness than on that occasion. The result of the operation was complete success."

His last operation was for cataract, which, from a record in his private Journal, seems also to have been his first. It was performed on the 13th of August, 1837, with a steady hand, while he was the subject himself of much mental and physical suffering. He never saw his patient after completing the operation, for the attack which terminated his laborious life occurred on the afternoon of the same day. On the morning of the 15th of December, at twenty minutes past 8 o'clock, the Father of American Surgery expired without a struggle. The immediate cause of his death was probably hydrothorax. For whole nights together he was unable to lie down in his bed, and was supported by assistants standing upon the floor. Anasarca was developed some time previous to his death, and in consequence of being obliged to stand so much upon his feet, his lower extremities became greatly swollen, the integuments gave way, and at length ulcerated and became gangrenous. He died in the 69th year of his age.

It is a little remarkable that he enjoined that no examination should be made of his body after death, and that a rigid watch should be kept over it for six weeks, during the night, to prevent disinterment.

"So protracted an illness," remarks Dr. Horner, "attended with such suffering, impaired also the vigor and clearness of his mind, and exhibited it not unfrequently in strong contrast with its natural traits. He left a paper directing the disposition of his body after death, as follows: a dissection was absolutely prohibited, no one was to touch him but two females who had been his domestics, for the last twenty years. He was not to be taken from his bed for some time, but to be wrapt up in it warmly; the room was to be kept well warmed till putrefaction commenced. He was then to be covered with flannel, and placed in a wooden coffin, painted outside, with a mattress at the bottom; and this coffin was to be placed within a leaden one, and it soldered up closely. A public notice was to be given of the period of his interment, but no invitation issued."

Dr. Horner's description of his appearance in the prime of life is graphic, and will be read with pleasure:

"Five and twenty years ago, I saw him who is now a mass of decomposition, returning from the same place in the afternoon at the interment of Dr. Rush. He was then in the vigor of manhood and of reputation, the universally acknowledged centre and head of the surgery of this country. An indescribable interval separated him from every body else, and yet attracted every one to him. I remember with perfect distinctness, as he turned off from the ground, his quick and thoughtful step; his inclination of the head: as either musing on what he had seen, or ruminating on some case of profound interest then under his charge. His appearance such as in his most palmy days—his head highly powdered; his hair overhanging his ears in a thick long brush on each side, where it was clipped straight below. The head, face, and neck, exhibiting the most finished and statue-like appearance; and his costume being a paragon of neatness, and of appropriateness, without any undue effort at effect. I never saw him before or since more completely himself. The attack of typhus fever, which he had the next winter, altered sensibly for the remainder of his life, the face—the forehead never recovered its fullness."

As a teacher of surgery Dr. Physick acquired a wide popularity; for the lectures of one whose experience had been so diversified could not be otherwise than instructive, while his character for integrity and strict veracity caused all to be believed which he professed to have done or seen. His manner is described as having been eminently dignified, grave and impressive, without being what is styled eloquent. He seems to have had little imagination, and to have indulged it sparingly. But he was judicious and tasteful; his style was clear, comprehensive, simple and concise. He wrote his lectures out with care, never trusting himself in extemporaneous discourse. His choice of language is represented as having been happy; his person was fine, and his whole air striking and agreeable; from which qualities it resulted that he was a most pleasing as well as instructive teacher. He attempted no display of oratory, but spoke clearly and forcibly from the stores of his memory and the dictates of his sound understanding.

If he had no great veneration for the recorded experience and learning of the profession, he had less disposition to give to the public the results of his own observation and experience; and the student of a future day will search in vain through the medical literature of our country for evidences of that superiority which while living was universally conceded to him. A description of a few surgical instruments invented or improved by himself, and brief histories of a few cases cured by novel modes of treatment, make up the sum total of all that his pen has contributed to the profession. His fame will rest upon what he did—not upon what he wrote. He was one of the first to make post mortem examinations; he introduced the operation of passing a seton between the ends of bones remaining long ununited; he introduced the animal ligature for arteries; he first recommended the use of blisters for the purpose of arresting the progress of mortification;—the excision of the uvula and of the tonsils in particular cases; the invention of several instruments, and the improvement of more—these are some of the achievements to which he was indebted for his great name. Y.

